# Post-Marketing Analysis of Peripheral Neuropathy Burden with New-Generation Proteasome Inhibitors Using the FDA Adverse Event Reporting System

**DOI:** 10.4274/tjh.galenos.2021.2021.0052

**Published:** 2021-08-25

**Authors:** Syeda A. Mina, Ibrahim N. Muhsen, Ethan A. Burns, Humaira Sarfraz, Sai Ravi Pingali, Jiaqiong Xu, Shahrukh K. Hashmi

**Affiliations:** 1 Mayo Clinic, Department of Medicine, Rochester, USA; 2 Houston Methodist Hospital, Department of Medicine, Houston, USA; 3 Houston Methodist Cancer Center, Houston Methodist Hospital, Houston, USA; 4 Center for Outcomes Research, Houston Methodist Research Institute, Houston, USA; 5 Mayo Clinic, Department of Medicine, Rochester, USA; 6 Sheikh Shakbout Medical City, Department of Medicine, Abu Dhabi, UAE

**Keywords:** Multiple myeloma, Proteasome inhibitors, Peripheral neuropathy

## Abstract

Proteasome inhibitors (PIs) are an integral component of multiple myeloma therapies. Peripheral neuropathy (PN) is a well-known consequence of PIs, most frequently reported with earlier generations such as bortezomib (BTZ). There is a paucity of data highlighting the risk of developing PN with the new-generation PIs carfilzomib (CFZ) and ixazomib (IZB). This study evaluated reports of PN encountered with all three PIs using the Food and Drug Administration Adverse Event (AE) Reporting System (FAERS). Signal disproportionality analysis was reported using the reporting odds ratio (ROR) with 95% confidence interval (CI). PN was reported in a total of 2.1%, 5.0%, and 10.9% of AEs with CFZ, IZB, and BTZ, respectively. The ROR (95% CI) for PN secondary to BTZ, CFZ, and IZB was 34.10 (32.76-35.49), 6.37 (5.50-7.37), and 14.97 (13.63-16.44), respectively. Compared to BTZ, CFZ and IZB have lower rates of reported PN, with RORs of 0.19 (0.16-0.22) and 0.48 (0.43-0.54), respectively.

## Abstract (tr)

Proteozom inhibitörleri (Pİ) multipl myelom tedavilerinin integral parçalarındandır. Çevresel nöropatiler (ÇN) Pİ’nin iyi bilinen bir sonucudur, bortezomib (BTZ) gibi önceki jenerasyonlarla daha sık bildirilmiştir. Yeni jenerasyon PI olan karfilzomib (KFZ) ve iksazomib (IZB) ile gelişen ÇN riskini vurgulayan çalışmalar yetersizdir. Bu çalışmada, Gıda ve İlaç İdaresi Olumsuz Olay (OO) Raporlama Sistemini (FAERS) kullanarak her üç PI ile karşılaşılan ÇN raporları değerlendirildi. Sinyal orantısızlık analizi, raporlama olasılık oranı (ROO) %95 güven aralığı (GA) kullanılarak yapıldı. ÇN; KFZ, IZB, BTZ ile sırasıyla OO’nun toplamında %2,1, %5,0, ve %10,9’unda rapor edilmişti. BTZ, KFZ, ve IZB’ye ikincil ÇN için ROO (%95 GA) sırasıyla 34,10 (32,76-35,49), 6,37 (5,50-7,37), ve 14,97 (13,63-16,44) idi. KFZ ve IZB ile, BTZ ile karşılaştırıldığında daha az ÇN rapor edilmişti, ROO sırasıyla 0,19 (0,16-0,22) ve 0,48 (0,43-0,54) idi.

## Introduction

Multiple myeloma (MM) is an incurable clonal plasma cell disease expected to be diagnosed in 34,920 individuals in the year 2021 in the United States alone [1]. While the incidence has remained stable over the past decade, the prevalence and 5-year overall survival (OS) rate have substantially improved with therapeutic breakthroughs in monoclonal antibodies, immunomodulatory therapy, and, recently, proteasome inhibitors (PIs) [2,3]. Currently, PIs are approved in combination with other agents in induction therapies for newly diagnosed MM, for autologous stem cell transplant candidates as maintenance therapies, and in cases of relapsed/refractory disease [2,4]. There are three PIs approved by the US Food and Drug Administration (FDA): bortezomib [(BTZ), approved in 2003], carfilzomib [(CFZ), approved in 2012], and ixazomib [(IZB), approved in 2015].

Despite profound therapeutic benefits, there are significant adverse events (AEs) that may impact the quality of life and impede medication use, one such toxicity being peripheral neuropathy (PN) [5]. This is most commonly reported with BTZ, and it has been reported as a dose-limiting toxicity in as many as 30%-60% of patients [6]. Recent clinical trials have suggested that the newer PIs have a lower association with these off-target effects [7,8]. However, there remains a relative paucity of post-marketing data on PN. This analysis aims to compare the rate of reported PN incidences with BTZ, CFZ, and IZB using the FDA Adverse Event Reporting System (FAERS).

## Materials and Methods

This study is a retrospective analysis using cases of AEs reported to the FAERS pharmacovigilance database. This public database is used by the FDA for post-marketing safety surveillance of pharmaceutical products. AEs and medication errors are coded to terms in the Medical Dictionary for Regulatory Activities. The cases are reported by healthcare professionals, manufacturers, and consumers. With input from 1968 to June 2020, the database contains approximately 19 million AE reports from around the world. Each reported event includes information about the constituents of the suspected product, indication for use, adverse reactions, characterization of the outcome (serious vs. non-serious, and whether hospitalization was required), patient demographics (gender, age, weight), and details of the reporting event (latest manufacturer report received to date, initial FDA report received, reporting country, and reporter type). All events have a unique case identification number.

This study retrospectively reviews AEs reported from the date of FDA approval of the respective medications, BTZ (2003), CFZ (2012), and IZB (2015), through June 20, 2020. The FAERS was queried for “bortezomib,” “ixazomib,” and “carfilzomib.” The following terms were subsequently selected as reasons for use: “plasma cell leukemia,” “plasma cell myeloma,” “plasma cell myeloma in remission,” and “plasma cell myeloma recurrent.” The specific AEs included were “peripheral neuropathy,” “sensory peripheral neuropathy,” “motor peripheral neuropathy,” and “sensorimotor peripheral neuropathy.”

### Analysis

Reported AEs attributed to new-generation PIs, namely CFZ and IZB, were compared individually to the total number of events reported to FAERS and to those for BTZ. Signal disproportionality analysis was performed by calculating the reporting odds ratio (ROR) and 95% confidence interval (CI). The ROR was reported to be significant if the 95% CI did not surpass 1, with an ROR of >1 suggestive of a higher likelihood of AEs being reported with the drug and <1 suggesting AEs to be less likely to occur with the drug. The chi-square test was used to calculate p-values, with p<0.05 being taken as significant. Continuous data were reported as means with standard deviations.

## Results

The number of adverse events of PN reported per year following the approval of the respective PIs are presented in Table 1. The mean patient age was 65.5±9.9, 67.7±10.1, and 68.5±9.5 years in the BTZ, CFZ, and IZB groups, respectively, with male-to-female ratios of 1.26:1, 1.3:1, and 1.01:1, respectively. The total numbers of PN events reported in the FAERS from the first FDA approval through June 2020 were 2802 (10.9%) for BTZ, 191 (2.06%) for CFZ, and 475 (4.98%) for IZB in comparison to the total reported AE rate in MM patients (Table 2). In most cases, PN was reported in the absence of reported concomitant medications, at rates of 71.8% for BTZ, 70.3% for IZB, and 51.8% for CFZ.

**Figure 1:**
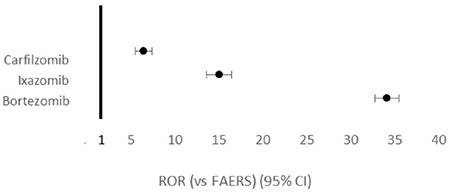
Forest plot of reporting odds ratios (RORs) with 95% confidence intervals (CIs) of adverse events reported for proteasome inhibitors compared to the Food and Drug Administration Adverse Event Reporting System (FAERS).

**Figure 2:**
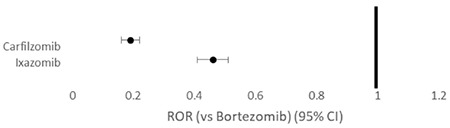
Forest plot of reporting odds ratios (RORs) with 95% confidence intervals (CIs) of adverse events reported for ixazomib and carfilzomib compared to bortezomib.

Compared to the entirety of the FAERS database for the corresponding years, the ROR (95% CI) for PN reported with BTZ, CFZ, and IZB was 34.10 (32.76-35.49), 6.37 (5.50-7.37), and 14.97 (13.63-16.44), respectively (Figure 1). When comparing CFZ and IZB to BTZ, there was a lower reported rate of PN, with RORs of 0.19 (0.16-0.22) and 0.48 (0.43-0.54), respectively, as shown in Figure 2.

## Discussion

The results of this study suggest a higher risk of reported PN with PIs, but when compared individually, there is lower risk with the new-generation PIs. PIs have become the standard of care in the treatment of MM. Recent data indicate higher levels of treatment-related toxicities with BTZ. For instance, the multicenter ENDEAVOR trial, which entailed a head-to-head comparison of BTZ versus CFZ in patients with relapsed or refractory MM, reported ≥ grade 2 PN in 32% of patients in the BTZ group compared to 6% in the CFZ group [9]. This analysis further substantiates these results and suggests that BTZ is more likely to be linked to reported PN than CFZ. Moreover, the ENDEAVOR and CLARION trials have consistently shown improved progression-free survival rates with CFZ that suggest it to be a more tolerable and potentially more efficacious option [9,10]. The present study also suggests a significantly lower likelihood of PN reported with IZB compared to BTZ.

Moreover, among the new-generation PIs, the post-marketing data showed a higher number of PN events reported to the FAERS with IZB versus CFZ despite later approval. During the safety analysis of single-agent CFZ in phase II clinical trials, the overall rate of PN was 13.9%. Furthermore, the majority of patients (87.3%) with baseline PN did not report worsening of PN while receiving CFZ [11]. On the contrary, the TOURMALINE-MM1 clinical trial showed a higher incidence of PN with the addition of IZB to a regimen of lenalidomide-dexamethasone (27% in the IZB group and 22% in the placebo group) [12]. This may suggest a higher propensity of developing PN with IZB. Although no head-to-head studies have compared AE profiles between the two newer-generation PIs, the potential for this treatment limiting AEs should be further substantiated in clinical analyses in the standard of care setting.

Although the FAERS database gives insight into important long-term trends associated with medications, some limitations should be recognized. It is a heterogeneous database that only allows for retrospective review. As such, treatment recommendations should not be derived from these findings, but this work does identify potential trends in toxicities that should be monitored and validated in additional studies. Additionally, the true incidence of an event occurring cannot be determined since the ratio of events occurring is based on total AEs rather than the true number of patients receiving medication. Moreover, the FAERS provides limited data about patients’ baseline comorbidities, thus introducing possible confounding factors that could serve as a predisposition to PN, including diabetes. Finally, PIs are typically given in combination with other myeloma agents that may predispose to PN. It is important to recognize that these patients were likely exposed to BTZ prior to trials of CFZ or IZB, as the new-generation PIs are rarely used as frontline therapy. It is possible that these agents further impacted the risk of PN development in these patients.

## Figures and Tables

**Table 1:**
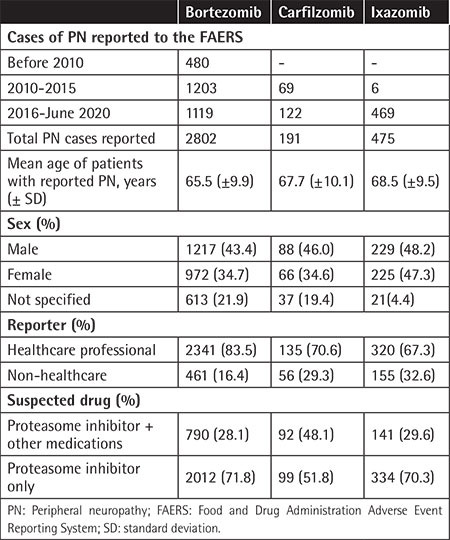
Summary of characteristics of patients with plasma cell disorders on proteasome inhibitors with side effects of peripheral neuropathy.

**Table 2:**
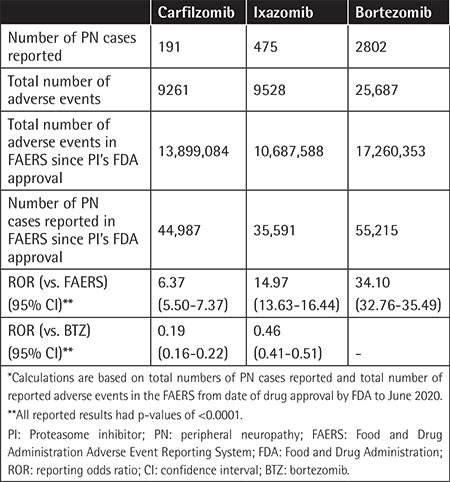
Comparative peripheral neuropathy reports in FAERS between different PIs.*
